# Prevalence and risk factors of colonisation with vancomycin-resistant Enterococci faecium upon admission to Germany’s largest university hospital

**DOI:** 10.3205/dgkh000377

**Published:** 2021-01-29

**Authors:** Minh Trang Bui, Anna M. Rohde, Frank Schwab, Nayana Märtin, Marina Kipnis, Anne-Cathérine Boldt, Michael Behnke, Luisa A. Denkel, Axel Kola, Janine Zweigner, Petra Gastmeier, Miriam Wiese-Posselt

**Affiliations:** 1 Charité – Universitätsmedizin Berlin, corporate member of Freie Universität Berlin, Humboldt-Universität zu Berlin, and Berlin Institute of Health; Institute of Hygiene and Environmental Medicine, Berlin, Germany; 2 German Center for Infection Research (DZIF), Braunschweig, Germany; 3 University Hospital Cologne, Department of Infection Control and Hygiene, Cologne, Germany

**Keywords:** vancomycin-resistant enterococcus, vancomycin-resistant E. faecium, admission prevalence, risk factors

## Abstract

**Background:** Hospital-acquired infections due to vancomycin-resistant enterococci (VRE) are emerging globally. The aims of our study were to estimate VRE colonisation prevalence in patients upon admission, to determine possible risk factors for VR *E. faecium* acquisition that already exist in the outpatient setting, and to monitor whether VRE-colonised patients developed a VRE infection during their current hospital stay.

**Methods:** In 2014 and 2015, patients admitted to non-intensive care units were screened for rectal VRE carriage. The study patients filled out a questionnaire on potential risk factors. Analyses were restricted to VR *E. faecium* carriage. All patients with VRE colonisation were retrospectively monitored for infections with VRE during their current hospital stay.

**Results:** In 4,013 enrolled patients, the VRE colonisation prevalence upon admission was 1.2% (n=48), and colonisation prevalence was 1.1% (n=45) for VR *E. faecium*. Only one VRE-colonised patient developed an infection with the detection of a VRE, among others. Colonisation with VR *E. faecium* was associated with current antibiotic use. Risk factors of VR *E. faecium* colonisation upon admission were increasing age, previous colonisation or infection with multidrug resistant organisms, sampling year 2015, and, within the previous six months, antibiotic exposure, a stay at a rehabilitation center, and a hospital stay.

**Conclusions:** We observed that antibiotic treatment which occurred prior admission influenced VR *E. faecium* prevalence upon admission. Thus, wise antibiotic use in outpatient settings plays a major role in the prevention of VR *E. faecium* acquisition.

## Zusammenfassung

**Hintergrund:** Krankenhausinfektionen durch Vancomycin-resistente Enterokokken (VRE) nehmen weltweit deutlich zu. Ziel unserer Studie war es, die Prävalenz der VRE-Kolonisation bei Patienten zum Zeitpunkt ihrer stationären Aufnahme zu bestimmen, mögliche Risikofaktoren für den Erwerb von VR *E. faecium*, die bereits im ambulanten Bereich bestehen können, zu beschreiben und nachzuverfolgen, ob VRE-kolonisierte Patienten während ihres aktuellen Krankenhausaufenthalts eine VRE-Infektion entwickeln.

**Methoden:** In den Jahren 2014 und 2015 wurden Patienten, die auf Nicht-Intensivstationen aufgenommen wurden, auf rektale VRE-Kolonisation untersucht. Die Studienpatienten füllten einen Fragebogen zu potenziellen Risikofaktoren aus. Nachfolgende Analysen wurden auf VR *E. faecium* Trägertum beschränkt. Alle Patienten mit VRE-Kolonisation wurden retrospektiv auf Infektionen mit VRE im Rahmen des aktuellen Krankenhausaufenthaltes nachverfolgt.

**Ergebnisse:** Bei 4.013 in die Studie aufgenommenen Patienten betrug die VRE-Kolonisationprävalenz bei der Aufnahme 1,2% (n=48) und die für VR *E. faecium* 1,1% (n=45). Nur ein Patient mit Nachweis von VRE bei Aufnahme entwickelte während des stationären Aufenthaltes eine Infektion mit Nachweis von VRE. Die VR* E. faecium*-Kolonisation war mit dem aktuellen Antibiotikaeinsatz assoziiert. Risikofaktoren für eine VR *E*. *faecium*-Kolonisation bei stationärer Aufnahme waren zunehmendes Alter, eine frühere Kolonisation oder Infektion mit multiresistenten Erregern, und Probenentnahme im Jahr 2015. Folgende Faktoren der letzten sechs Monate waren zudem mit VR *E. faecium*-Kolonisation assoziiert: Antibiotikaeinnahme, Aufenthalt in einem Rehabilitationszentrum und Krankenhausaufenthalt.

**Schlussfolgerungen:** Wir beobachteten, dass eine antibiotische Behandlung, die vor der stationären Aufnahme erfolgte, die VR *E. faecium*-Prävalenz bei Aufnahme beeinflusste. Daraus kann geschlossen werden, dass der weise Einsatz von Antibiotika im ambulanten Versorgungsbereich eine wichtige Rolle bei der Prävention von VR* E. faecium*-Kolonisation spielt.

## Introduction

Hospital-acquired (HA) infections due to vancomycin-resistant enterococci (VRE) are increasing globally. In 2018, the World Health Organization (WHO) ranked vancomycin-resistant (VR) *Enterococcus (E.) faecium* as a pathogen of high priority for research and development of effective drugs [1]. The results of a point prevalence survey of HA infections in Germany in 2016 showed that, of 1,817 patients with HA infection and concurrent pathogen detection, *E. faecalis* was identified in 6.9% of the cases and* E. faecium* in 5.7% [2]. In Europe, the population-weighted mean percentage for vancomycin resistance in *E. faecium* isolates increased from 10.4% in 2014 to 17.3% in 2018 [3]. Nosocomial dissemination of VRE, as well as antibiotic treatment, have been discussed as reasons for HA VRE infections. Data on antibiotic use at our hospital suggests that the risk of HA VRE detection in clinical samples is associated with the use of glycopeptide or carbapenem antibiotics [4]. Patients infected with VRE or patients merely colonised by VRE were considered the source of transmission in hospital outbreaks [5], [6], [7]. Nevertheless, reducing infection-control measures in VRE colonised patients, such as single-room contact isolation or the use of personal protective clothing by healthcare staff in hospitals, has been discussed since the infection rate in VRE colonised patients is low and the impact of VRE colonisation or infection on mortality remains unclear [8], [9], [10]. Moreover, other risk factors for VRE colonisation and infection have been considered, such as prolonged hospital stay, underlying diseases or comorbidities (e.g. cancer, diabetes mellitus, and renal failure), and immuno-suppression [4], [11], [12], [13]. Public health authorities have emphasised the need for better knowledge on the epidemiology of VRE in order to develop prevention and infection control strategies [1] , [3]. 

To address that need, our study aimed to estimate VRE colonisation prevalence in patients upon admission and to determine possible risk factors for VRE or VR *E. faecium* colonisation that already exist prior to hospitalisation. Patient follow-up was performed to monitor subsequent VRE infections and to identify possible influencing factors. In recent years, *E. faecium* in particular has rapidly developed into a worldwide nosocomial pathogen, because it has successfully adapted to conditions in a nosocomial environment and has acquired resistance to glycopeptides [14]. Therefore, we focused our risk analysis on VR *E. faecium*.

## Methods

### Setting

Our study is based on a sub-population of the Antibiotic Therapy Optimization Study (ATHOS), which was conducted in six German university hospitals from January 1, 2014 to December 31, 2015 [15], [16]. The Charité is one of Europe’s largest university hospitals with about 3,000 beds distributed over three separate clinic sites. In addition to the ATHOS-wide admission prevalence screening, at Charité University Clinic, we performed patient follow-up to assess subsequent VRE infection. 

The study was approved by the ethics committee of the Charité University Hospital of Berlin under the approval number EA4/018/14. Patients granted informed consent prior to inclusion.

### Participants, questionnaire, and follow-up 

Adult patients (18 years or older) who had been admitted to non-intensive care units (non-ICUs) at all three sites of Charité University Hospital were screened for rectal colonisation of multidrug resistant organisms (MDRO) and followed-up for VRE infection. Patients hospitalised in ICUs or general psychiatric, dermatology, otorhinolaryngology, ophthalmology, paediatric and obstetric wards were excluded from the study [16], [17]. 

The enrollment methodology has been described previously [16]. In brief, rectal swabs were taken within three days of admission (day of admission=day 1) either by the healthcare staff or under supervision by the patients themselves. In addition, each patient answered a questionnaire on potential risk factors (see Attachment 1 Appendix A for questionnaire) [16]. Data on place of residence (district of Berlin), nationality classified by WHO region, ward of admission, acquisition of an infection during the current hospital stay, and anamnestic data as defined by the Charlson comorbidity index (CCI) were extracted from electronic patient files [18]. 

### Laboratory methods 

Rectal samples were transferred onto a blood agar plate (Columbia Agar +5% sheep blood, bioMérieux, Nürtingen, Germany) as well as a ChromID VRE agar plate (bioMérieux, Nürtingen, Germany). All pathogens cultivated on selective culture media were identified down to species level using VITEK2 (bioMérieux, Nürtingen, Germany). Antimicrobial susceptibility testing was also performed with VITEK2. Enterococci were classified as susceptible or resistant to glycopeptides based on minimal inhibitory concentrations according to the breakpoints of the European Committee on Antimicrobial Susceptibility Testing (EUCAST) [19], [20]. Non-susceptibility was regarded as resistance. Very experienced laboratory personnel performed the laboratory testing. Any conflicting results were resolved by multiplex VanA/B-PCR [20], [21].

### Definitions 

Patients who tested positive for VRE in their rectal samples upon admission and with absence of VRE in a clinical specimen (e.g., urine or blood) were defined as VRE colonised. The case was defined as a VRE infection if the electronic patient files reported a patient’s clinical specimen to be positive for VRE upon admission or VRE were detected in a clinical specimen during the current hospital stay, with additional signs and symptoms of infection as determined by a clinician and followed by adequate antimicrobial therapy.

### Statistical analysis 

The number of patients who tested positive for VRE and VR *E. faecium* per 100 patients screened upon admission defined the VRE and VR *E. faecium* prevalence rate, respectively. In the descriptive analysis, numbers and percentages were calculated as well as probability values using the Chi-squared or Fisher’s exact test. The CCI is only reported descriptively. In the multivariable analysis, logistic regression models were applied to identify risk factors for colonisation by VR *E. faecium* upon admission. The following patient-based parameters were considered in the analyses: sex (male/female); age (≤45, 46–55, 56–65, 66–75 or >75); prior MDRO (methicillin-resistant *Staphylococcus aureus* (MRSA), third-generation cephalosporin-resistant enterobacteriaceae (3GCRE), carbapenem-resistant enterobacteriaceae, and/or VRE) colonisation/infection; current antibiotic use, antibiotic use in the previous six months; travel abroad in the previous six months inside or outside Europe; a stay at a rehabilitation center or long-term care facility (LTCF) during the previous six months; a hospital stay during the previous six months; occupational or private contact with animals; and treatment of gastroesophageal reflux disease (GERD) with antacids or proton-pump inhibitors during the previous six months. Parameters were categorised as “no” (reference), “yes” or “unknown.” In multivariable analysis, the category “unknown” was allocated to “no”. The variable “ward of admission” was grouped as described in the Attachment 2, Appendix B. 

The following parameters, which were identified by means of the electronic patient files, were included as binary variables: place of residence (unknown, not Berlin, and the districts of Berlin (Attachment 2, Appendix B)) and nationality classified by WHO region (Attachment 2, Appendix B). 

In the multivariable analysis, the model-building strategy was performed stepwise backward, and the significance level for excluding a parameter from the model was p=0.05. For epidemiological reasons, age and sex were included in all models. P-values <0.05 were considered significant. All analyses were performed using SPSS 22 (IBM SPSS Statistics, Armonk, NY, USA) and SAS 9.3 (SAS Institute, Cary, NC, USA). 

## Results

### Study participants 

**Figure 1:**
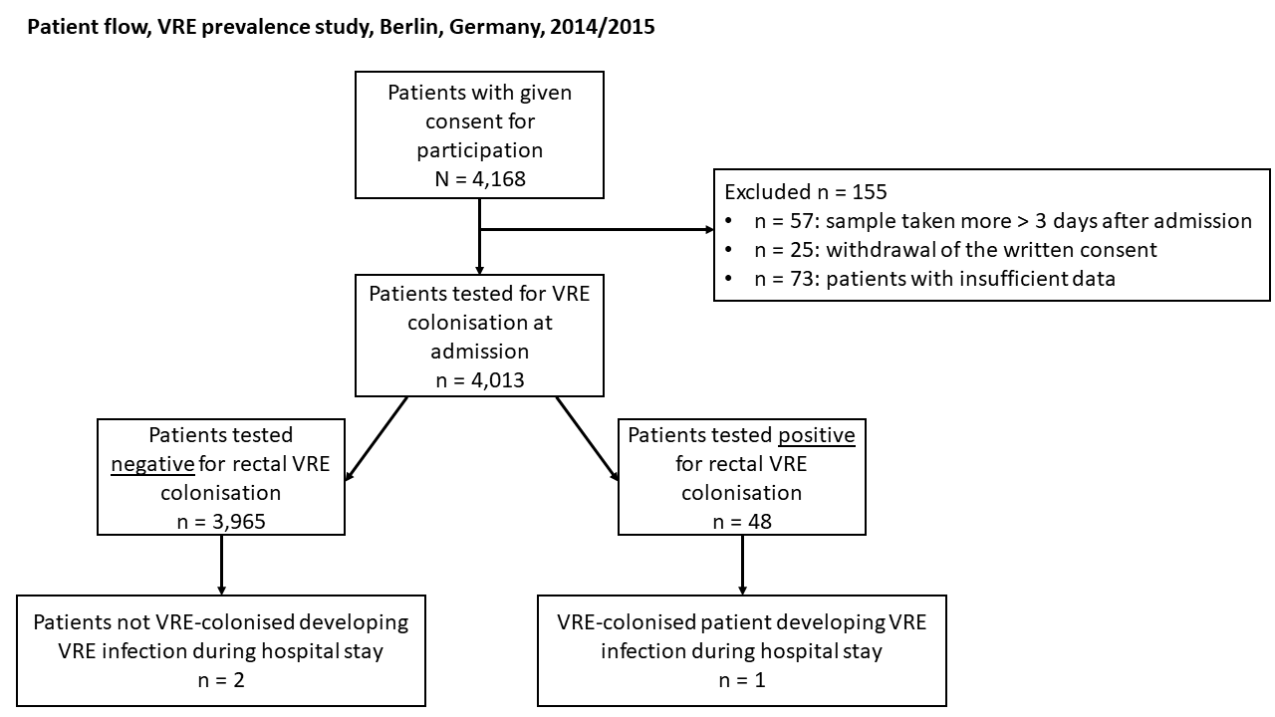
Patient flow, VRE prevalence study, Berlin, Germany, 2014/2015

In the study periods in 2014 and 2015, 4,013 patients total were included in the prevalence study. Initially, 4,168 patients enrolled. However, because of a sample taken more than three days after admission (n=57), withdrawal of the written consent of 25 participants, and patients with insufficient data (n=73), 155 participants dropped out of our study cohort (see Figure 1 (Fig. 1)). Of 4,013 patients, 2,007 (50.0%) were female. Median age was 62 years (inter quartile rage (IQR 50–73). The CCI, available for 3,900 patients (97.1%), was three (IQR 1–5).

### Patients with VRE colonisation upon admission 

VRE colonisation prevalence upon admission was 1.2 per 100 patients screened (n=48). In four patients, 3GCRE was also detected. Of the 48 VRE samples, *E. faecium* was detected in 45 (93.75%) and *E. faecalis* in three (6.25%). The median age of VRE-colonised patients was 71 years (IQR 59-75.5). Of these, 22 patients (46%) were female. The CCI in VRE-colonised patients was 5 (IQR 3-7); however, in 6 of 48 patients, the CCI was not available. Therefore, CCI was not considered in further analyses. 

### VRE infections during the hospital stay 

Only one patient who was VRE colonised upon admission developed a polymicrobial wound infection from a vancomycin-resistant strain of *E. faecium*, non-resistant *E. faecium*, *Streptococcus sanguinis*, and *Klebsiella oxytoca* (Figure 1 (Fig. 1)). Unfortunately, a genome analysis and comparison of the VRE strain upon admission and the VRE strain causing the wound infection were not possible. Two patients without rectal VRE detection upon admission developed a VRE infection during their hospital stay.

### Risk factors of VR E. faecium colonisation at admission 

As already mentioned, we restricted the descriptive and risk factor analyses on VR *E. faecium*. Data from the descriptive analysis of patient demographics is shown in Table 1 (Tab. 1); data on possible risk factors recorded in the patients’ questionnaire is given in Table 2 (Tab. 2). The frequency of VR *E. faecium* colonisation was not associated with sex. However, it increased with age. Moreover, admission to a haematology/oncology ward (VR *E. faecium* prevalence per 100 patients 2.7, p-value (p) 0.038) or a radiation therapy ward (5.0, p 0.019), a rectal probe taken in 2015 as opposed to 2014 (prevalence 1.5 versus 0.8, p=0.27), and place of residence in the Berlin district Reinickendorf (2.9, p=0.006) or unknown place of residence (9.1, p=0.012) were significantly associated with the occurrence of rectal VR *E. faecium* (Table 1 (Tab. 1)). 

Based on the data from the questionnaires, the following parameters were identified as possible risk factors: current antibiotic use (at the time of the questionnaire), previous colonisation or infection with MDRO, and – in the previous six months – antibiotic exposure, travelling abroad, a stay at a rehabilitation center or hospital, and treatment for GERD (Table 2 (Tab. 2)).

The results of the multivariable logistic regression model are shown in Table 3 (Tab. 3). Age 45 or younger and being a resident of the Berlin district Steglitz-Zehlendorf seemed to lower the likelihood for VR *E. faecium* colonisation upon admission. Moreover, a high odds ratio (OR) of 11.90 for VR *E. faecium* colonisation was reported for patients whose place of residence was unknown. However, there is a very broad 95% confidence interval (CI) of 1.12 to 126.99. Current exposure to antibiotics as well as during the previous six months and prior to MDRO colonisation or infection, regardless of the species, were confirmed as risk factors for VR *E. faecium* acquisition. Furthermore, a stay at a rehabilitation center and a hospital during the previous six months was identified as a risk factor. Sampling the rectal probes in the year 2015 as opposed to 2014 continued to be significantly associated with rectal VR *E. faecium* detection in the multivariable analysis (Table 3 (Tab. 3)). 

## Discussion

In Germany, VR *E. faecium* infections are increasing [22]. Data from the German Antimicrobial Resistance Surveillance System show a continuous increase of the proportion of VR *E. faecium* in clinical material isolates from 11.2% (95% CI 9.4–13.3%) in 2014 to 26.1% (95% CI 23.1–29.4%) in 2017 [22]. Thus, the German rate of vancomycin resistance in *E. faecium* exceeded the overall mean rate of 17.3% in Europe [3], [23]. This epidemiological VRE data is based on clinical invasive samples of hospitalised patients. In our study, we report prevalence data on VRE colonisation upon admission to non-ICUs, which until now rarely appear in published data. Recently, a Danish study on MDRO (including VRE) prevalence in patients who were admitted to an emergency department (ED) reported a VRE colonisation prevalence of 0.4 per 100 patients tested [24], which is even lower than the prevalence of 1.2 determined in our study. The lower VRE prevalence in Denmark can be explained by a lower level of vancomycin resistance in enterococci of clinical invasive samples of hospitalised patients. In 2018, vancomycin resistance in *E. faecium* was 12.5% in Denmark compared to 23.8% in Germany. In Ireland, a VRE prevalence of 22% to 31% among in-patients (n=121) and no VRE detection in an outpatient setting of general practitioners (n=29) was reported [25]. In a population-based study from the Netherlands, only one person out of 1,992 screened was carrying a VRE [26]. Thus, we can suggest that patients are more likely to acquire VRE in the hospital than in an outpatient setting. 

In our study, we looked for possible risk factors for VR *E. faecium* colonisation prior to hospitalisation. In the multivariable logistic regression, we identified well-known risk factors for VR *E. faecium* infection and colonisation (see Table 3 (Tab. 3)) [24]. In a Korean study, patients transferred from a geriatric long-term care hospital to the ED had a higher risk of VR *E. faecium* colonisation than patients who were transferred from a general hospital (OR of 8.0) [27]. However, in a study conducted in Singapore, the VRE prevalence was higher in patients in acute-care hospitals (14.2%) than in patients in long-term care facilities (0.8%) [28]. This heterogeneous pattern might be deceptive, because the risk of VRE acquisition does not seem to be associated with the type of healthcare facility but with the clinical index or health status of the patients themselves. Thus, the kind of hospital or department can function as a proxy, but the interpretation must be considered carefully. In respect to the CCI of our study patients, we can assume that a higher CCI is associated with VRE positivity upon admission. However, as already mentioned, in six of 48 VRE-colonised patients, the CCI was not available; thus, CCI was not entered as a variable in the univariable und multivariable analyses. Nevertheless, very ill and immunocompromised patients must receive the most vigilant care to prevent VRE colonisation and infection [29]. VRE surveillance data from European Union member states shows high variability in the rate of VR *E. faecium*, from 10% to almost 50% (in clinical samples). Lower VRE prevalence rates than from Germany are reported in France, Spain, and the Scandinavian countries, higher VRE prevalence in the United Kingdom, Greece, and some eastern EU countries [3]. This diverse epidemiological picture of VRE is found in Germany as well. Higher VRE and VR *E. faecium* prevalence rates were reported in the central German federal states than in the north or south of Germany [22], [30]. There is no clear explanation for these regional differences. One cause might be the regional emergence of specific VRE strains. In clinical and surveillance samples from different wards at Charité University Hospital from 2008 to 2018, a significant increase in VRE strain sequence type (ST)117 cluster type (CT)71 was reported [31]. Therefore, we can assume that the rising VRE prevalence at Charité University Hospital is due to strain ST177 CT71. Based on data on antimicrobial use density in Germany, some federal states with high VRE prevalence shown an antimicrobial use density above the German mean. Other federal states did not [31], [32]. Thus, the regional differences in the rate of VRE or VR *E. faecium* prevalence in Germany cannot be explained by the nationwide distribution of antimicrobial use density. 

It has been reported that even in high-income countries like Germany, the risk of illness is significantly higher for persons of low socioeconomic status (SES) than persons of medium or high SES [33]. In our study, patients who were residents of the district Steglitz-Zehlendorf showed a significantly lower VR *E. faecium* prevalence upon admission. This district is described as a region of Berlin with higher SES than reported from most other Berlin districts [34]. Therefore, we can suggest that a high SES might be associated with a lower VR *E. faecium* colonisation rate prior to hospitalisation. However, this is only a cautious assumption, as this is not a sociological study.

We detected a significant difference in VR *E. faecium* prevalence in patients sampled in the years 2014 or 2015. The methodology and the process of sampling in our study did not differ from year to year. The significantly higher VR *E. faecium* prevalence of 1.5 per 100 patients screened in 2015 compared to 0.8 in 2014 can be explained by the dynamics of VR *E. faecium* epidemiology in the last few years. As reported, at Charité University Hospital, the number of isolates representing the ST117 CT71 strain increased by 36.7% (p<0.001) [31]. German national nosocomial infection surveillance system (KISS) data confirms a trend of increasing VRE positivity in hospitalised patients between 2008 and 2016 that is in accordance with European surveillance data [3], [35]. An ecological study of antibiotic use at Charité University Hospital of Berlin showed that the rate of HA VRE isolates is associated with the use of certain antimicrobial agents, e.g., glycopeptide or carbapenem antibiotics [4]. The prescription of glycopeptides or carbapenems in outpatients is negligible, but the majority of our VR *E. faecium*-colonised patients had been hospitalised in the previous six months (n=30 in Germany, n=5 abroad) [36]. Therefore, a decisive role might be played by the use of glycopeptides or carbapenems during previous hospitalisations or by treatment with other antibiotics such as flucloxacillin or piperacillin/tazobactam, which are also reported to be associated with VRE acquisition [37]. In 2014, the total antimicrobial consumption density was 65.93 DDD (daily defined doses)/100 patient days (pd) at Charité University Hospital. It increased to 71.94 DDD/100 pd in 2015 and decreased in the following years [38]. On the other hand, VRE rates in patients hospitalised in ICUs in German hospitals continue to rise [39], [40]. Thus, VRE detection is significantly associated with antibiotic use; however, the sustained increase of VRE, especially VR *E. faecium*, must be influenced by other factors as well. 

### Strengths and limitations 

The present study has several strengths. For instance, it included a large sample size of over 4,000 patients from three separate clinic sites. We conducted the rectal screening of the patients upon their admission to non-ICUs and tracked them for VRE infection during their hospital stay. VRE or VR *E. faecium* prevalence rates upon admission are to date rarely reported. We identified possible risk factors of VR *E. faecium* colonisation that appear on a local or regional level and can be applied to other settings world-wide. We also investigated whether we could find evidence that colonised patients were more likely to develop an infection. 

Our study also has several limitations. First, we based our risk factor analysis on the self-reported information of the patients, which could bias the results of the risk factor analysis [41]. To minimise the influence of bias, the questionnaire was tailored to the perceptions of the patients. If they requested not to fill out the questionnaire on their own, study personnel completed it in interview mode. As this was not a social-epidemiological study, we have to look very carefully at the significantly low VR *E. faecium* prevalence among the inhabitants of the Berlin district Steglitz-Zehlendorf. Second, we allowed the patients to perform the rectal sampling themselves. However, the health care staff assisted them or took the rectal sample from the patient. In rectal specimens, the sensitivity for the detection of VRE is reported to be around 60% and it is determined by the concentration of VRE in the patient’s stool [42]. Sensitivity rises if repeated rectal sampling is performed [43]. Moreover, it is reported that the VITEK2 (bioMérieux, Nürtingen, Germany) is less sensitive than other methods in the performance of susceptibility testing. However, in case of conflicting results, we verified these by multiplex VanA/B-PCR. Nevertheless, it is possible that we underestimated the real VRE and VR *E. faecium* colonisation prevalence upon admission. Third, we report data of a large university hospital consisting of three separate clinic sites. Thus, the generalisability of our results to non-university hospitals (tertiary care) is limited. Regional differences in VRE epidemiology in Germany have been described elsewhere [30]. However, based on the median CCI in our patient cohort and data on CCI of other non-ICU patient cohorts in German university hospitals, we suggest that our results on risk factors and the general trend in VRE epidemiology are comparable with the situation of other non-ICU patient cohorts at German university hospitals [16]. 

## Conclusions

We determined a low prevalence rate of 1.2% in patients upon admission to non-ICUs. The increase of VRE and VR *E. faecium* prevalence from 2014 to 2015 is in line with the European and global trend of VRE epidemiology. In regard to VR *E. faecium*, we identified well-known risk and influencing factors such as antibiotic use even prior admission. Thus, wise antibiotic use in both the in-patient and the outpatient setting plays a major role in the prevention of VR* E. faecium* acquisition. 

## Notes

### Competing interests

The authors declare that they have no competing interests.

### Acknowledgements 

We would like to thank Solvy Wolke for study assistance and Gerald Brennan for proofreading.

### Author contributions

PG and JZ developed the study design and methodology. MTB, NM, MK, ACB collected the data. AK conducted microbiological analysis. FS conducted the analysis. AMR, LAD, MWP did project administration and validation. MTB validated data and was supervised by PG and MWP. MB provided input into study execution. The first draft of the manuscript was written by MTB and MWP. Subsequent versions were reviewed and edited by all co-authors. All authors read and approved the final manuscript.

### Financial support

The admission screening was done as part of the multi-center Antibiotic Therapy Optimization Study (ATHOS) supported by the German Center for Infection Research (DZIF). AMR and MWP were supported by DZIF (grant number TTU08.801, http://www.dzif.de). The funders had no role in study design, data collection and analysis, decision to publish, or preparation of the manuscript.

### Availability of data and material

Patient data used in this study is confidential according to the data privacy act and the institutional ethics committee of Charité Universitätsmedizin Berlin. In addition, information on location, ward of admission, age, sex, place of residence and nationality are indirect identifiers and might enable an interested researcher to trace the identity of the patient. To protect patient confidentiality and participant's privacy, data used for this study can be obtained in an anonymous and condensed form only according to the data privacy act. Interested researchers may contact it-hygiene@charite.de to receive access to the anonymized data used in this analysis, if approved by the data access committee at the “Institut für Hygiene und Umweltmedizin, Charité Universitätsmedizin Berlin”.

## Supplementary Material

Appendix A, questionnaire: case report of ATHOS prevalence study (page 1, filled in by the study personnel) and uestionnaire on risk factors for colonisation with MDRO (page 2, filled in by the study participants or completed by the study personnel in interview mode)

Appendix B, supplement to the information in the methods section

## Figures and Tables

**Table 1:**
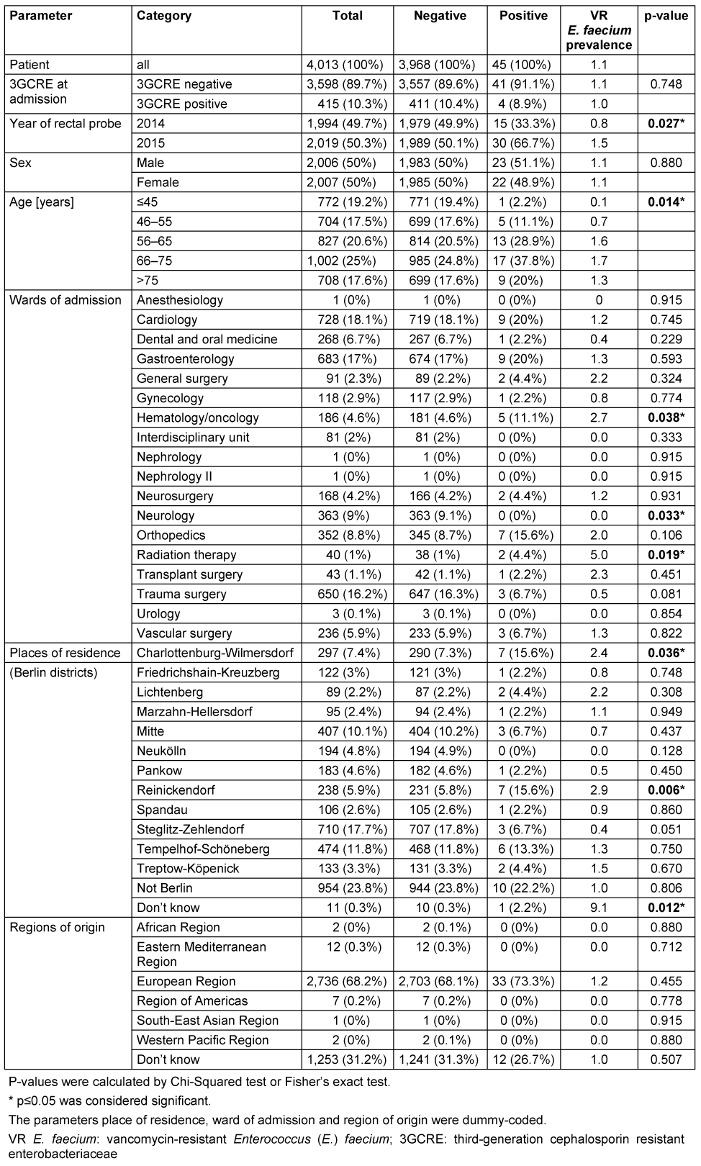
Descriptive analysis of demographic data of 4,013 patients screened for VRE colonisation upon admission to Germany’s largest university hospital; patients stratified by positive or negative VR *E. faecium* status; VRE prevalence study, Berlin, Germany, 2014/2015

**Table 2:**
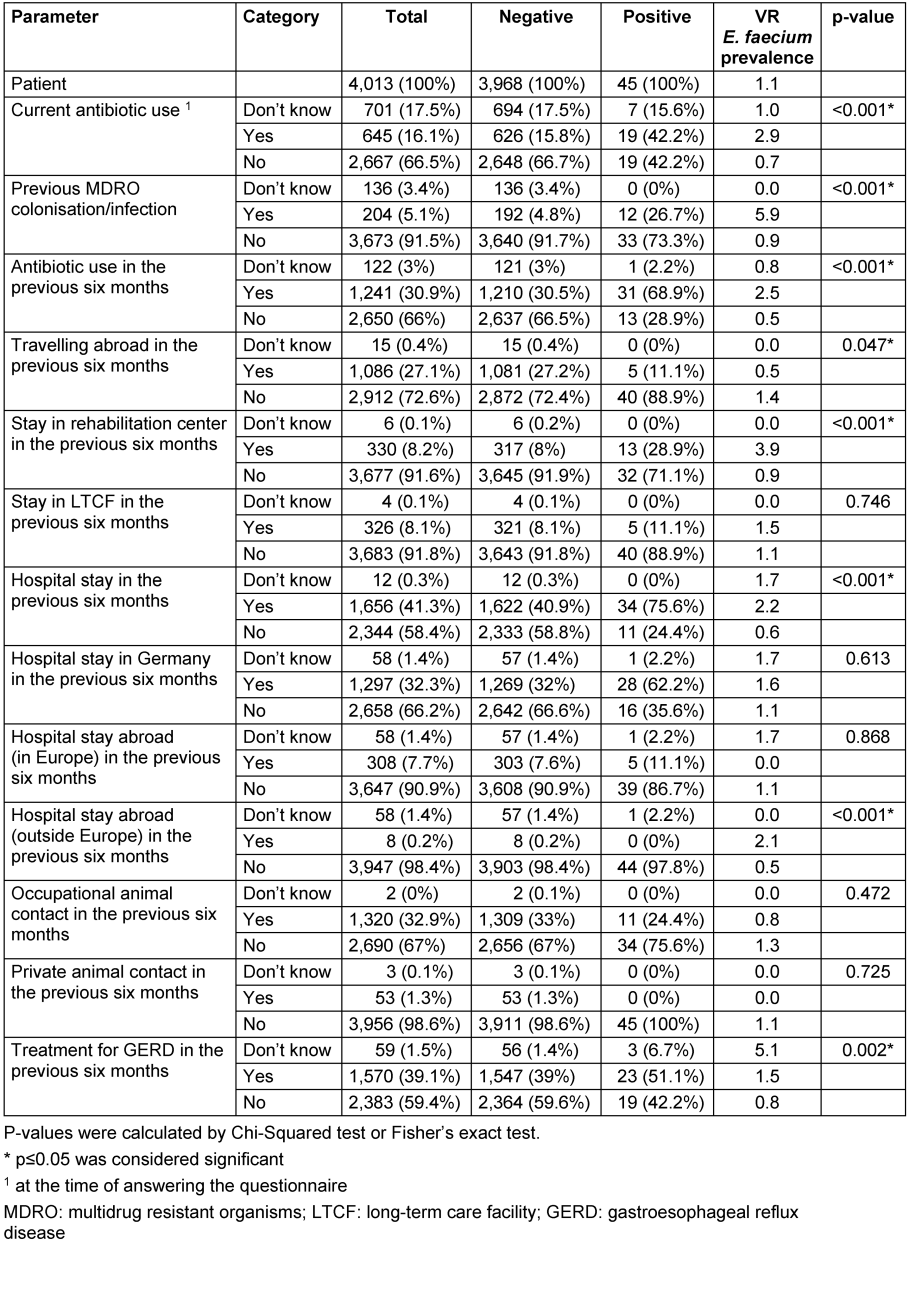
Descriptive analysis of possible risk factors in 4,013 patients screened for VRE colonisation upon admission to Germany’s largest university hospital; patients stratified by positive or negative VR *E. faecium status*; VRE prevalence study, Berlin, Germany, 2014/2015

**Table 3:**
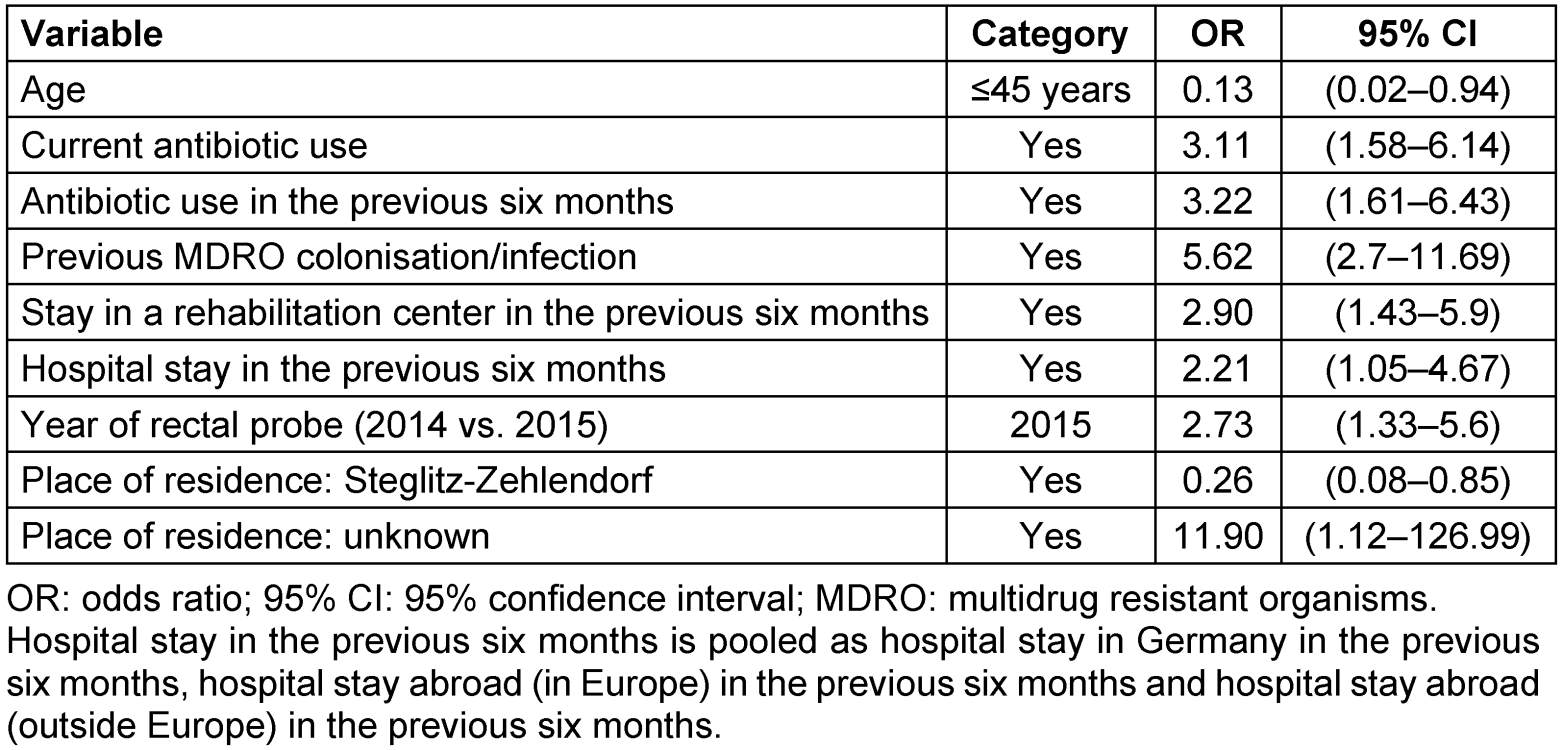
Results of the logistic regression model on risk factors for VR *E. faecium* colonisation upon admission to Germany’s largest university hospital, n=4,013 participating patients; VRE prevalence study, Berlin, Germany, 2014/2015
