# Supplementary material for: Prevalence and risk factors of colonisation with vancomycin-resistant Enterococci faecium upon admission to Germany’s largest university hospital
Source: GMS Hyg Infect Control. 2021 Jan 29;16:Doc06. doi: 10.3205/dgkh000377 (PMC7894188; doi:10.3205/dgkh000377)
Supplement: Appendix A, questionnaire: case report of ATHOS prevalence study (page 1, filled in by the study personnel) and uestionnaire on risk factors for colonisation with MDRO (page 2, filled in by the study participants or completed by the study personnel in interview mode) [file HIC-16-06-s-001.pdf]

Deutsches Zentrum für Infektionsforschung

Put Pat.ID sticker here

ATHOS prevalence study

Recruiter initials

A. Patient information

**Patient- ID (Studynumber)**					
Unit-ID		Age in years		Sex ❐ M	❐ F

**Entry date to ATHOS-Area (DD.MM.YY)**	
**Entry date to ATHOS-Unit (DD.MM.YY)**	
**Date of rectal swab (DD.MM.YY)**	
**Current AB therapy (oral/iv)?** ❐ **Yes** ❐ **No** ❐ **Unknown**	

B. Results of Screening

Negative ❐

Positive ❐

**Microbiological Finding** (resistant *Citrobacter*, *Escherichia*, *Enterobacter*, *Klebsiella*, *Salmonella*, *Serratia*, *Proteus* and *Hafnia* species)			
**Isolate 1** (genus + species)		**Isolate 2** (genus + species)	
**MDR-GN**	❐**3GCREB** ❐**3MDR-GN** ❐**4MDR-GN**	**MDR-GN**	❐**3GCREB** ❐**3MDR-GN** ❐**4MDR-GN**
**VRE**	❐**Yes** ❐**No**	**VRE**	❐**Yes** ❐**No**
**ESBL** (omit if VRE)	❐**Positive** ❐**Negative**	**ESBL** (omit if VRE)	❐**Positive** ❐**Negative**
**Colony number**	❐ **+** ❐ **++** ❐ **+++**	**Colony number**	❐ **+** ❐ **++** ❐ **+++**

Attachment 1 to: Bui MT, Rohde AM, Schwab F, Märtin N, Kipnis M, Boldt AC, Behnke M, Denkel LA, Kola A, Zweigner J, Gastmeier P, Wiese-Posselt M. Prevalence and risk factors of colonisation with vancomycin-resistant Enterococci faecium upon admission to Germany's largest university hospital. GMS Hyg Infect Control. 2020;16:Doc06. DOI: 10.3205/dgkh000377

Risk factor questionnaire

1. Were you ever diagnosed with a multidrug-resistant organism (colonisation or infection)?

❐ **Yes** ❐ **No** ❐ **Don't know**

If yes, which type? ❐MRSA ❐VRE ❐ESBL- Producer ❐3GCREB ❐3MDR-GN ❐4MDR-GN ❐Unknown

2. Did you take antibiotics in the previous 6 months (not including a current AB Therapy)?

❐ **Yes** ❐ **No** ❐ **Don't know**

3. Have you been abroad in the last 6 months?

❐ **Yes** ❐ **No** ❐ **Don't know**

If yes, make up to three entries: **<u>If in Europe, indicate name of country</u>** and **<u>if outside Europe, indicate the region</u>**:

______________________ ______________________ ______________________

❐ Africa ❐Asia (if in India, indicate: ❐) ❐North America ❐ Central and South Amerika
❐Australia + Oceania ❐Arabian Peninsula

4. Have you been in a rehabilitation facility during the last 6 months?

❐ **Yes** ❐ **No** ❐ **Don't know**

5. Have you been in a long-term care facility during the last 6 months?

❐ **Yes** ❐ **No** ❐ **Don't know**

6. Have you been in a hospital <u>in Germany or abroad</u> for in-patient care during the last 6 months?

❐ **Yes** ❐ **No** ❐ **Don't know**

If yes, make up to three entries: **<u>If in Europe, indicate name of country</u>** and **<u>if outside Europe, indicate the region</u>**:

______________________ ______________________ ______________________

❐ Africa ❐Asia (if in India, indicate: ❐) ❐North Amerika ❐ Central and South Amerika
❐Australia + Oceania ❐Arabian Peninsula

7. Do you have contact with animals as part of your job?

❐ **Yes** ❐ **No** ❐ **Don't know**

8. Do you have pets?

❐ **Yes** ❐ **No** ❐ **Don't know**

9. Have you taken medication for gastroesophageal reflux disease during the last 6 months?

❐ **Yes** ❐ **No** ❐ **Don't know**

Medication against gastroesophageal reflux disease are e.g.:
a. Antacids
b. Proton-pump inhibitors

Attachment 1 to: Bui MT, Rohde AM, Schwab F, Märtin N, Kipnis M, Boldt AC, Behnke M, Denkel LA, Kola A, Zweigner J, Gastmeier P, Wiese-Posselt M. Prevalence and risk factors of colonisation with vancomycin-resistant Enterococci faecium upon admission to Germany's largest university hospital. GMS Hyg Infect Control. 2020;16:Doc06. DOI: 10.3205/dgkh000377
