# Supplementary material for: Prevalence and risk factors of colonisation with vancomycin-resistant Enterococci faecium upon admission to Germany’s largest university hospital
Source: GMS Hyg Infect Control. 2021 Jan 29;16:Doc06. doi: 10.3205/dgkh000377 (PMC7894188; doi:10.3205/dgkh000377)
Supplement: Appendix B, supplement to the information in the methods section [file HIC-16-06-s-002.pdf]

Appendix B

Supplements to the information in the methods section

1.) The variable “ward of admission” was grouped as followed:

- The departments of haematology/oncology and radiation therapy were merged into the ward of admission “HAEMA”.
- All surgical departments were merged into “SURG”.
- Internal medicine, gynaecology, neurology, and interdisciplinary units were merged into “MED”.
- Due to small patient counts, anaesthesiology, urology, and nephrology were classified together as “Other wards”.

2.) The districts of Berlin are Charlottenburg-Wilmersdorf, Friedrichshain-Kreuzberg, Lichtenberg, Marzahn-Hellersdorf, Mitte, Neukölln, Pankow, Reinickendorf, Steglitz-Zehlendorf, Spandau, Tempelhof-Schöneberg, and Treptow-Köpenick.

3.) The WHO regions are African Region, Region of Americas, South-East Asia Region, European Region, Eastern Mediterranean Region and Western Pacific Region.

Attachment 2 to: Bui MT, Rohde AM, Schwab F, Märtin N, Kipnis M, Boldt AC, Behnke M, Denkel LA, Kola A, Zweigner J, Gastmeier P, Wiese-Posselt M. Prevalence and risk factors of colonisation with vancomycin-resistant Enterococci faecium upon admission to Germany’s largest university hospital. GMS Hyg Infect Control. 2020;16:Doc06. DOI: 10.3205/dgkh000377
